# TAFRO Syndrome with Renal Thrombotic Microangiopathy: Insights into the Molecular Mechanism and Treatment Opportunities

**DOI:** 10.3390/ijms22126286

**Published:** 2021-06-11

**Authors:** Kun-Hua Tu, Pei-Yi Fan, Tai-Di Chen, Wen-Yu Chuang, Chao-Yi Wu, Cheng-Lung Ku, Ya-Chung Tian, Chih-Wei Yang, Ji-Tseng Fang, Huang-Yu Yang

**Affiliations:** 1 Kidney Research Center, Department of Nephrology, Chang-Gung Memorial Hospital, Taoyuan 333, Taiwan; 8902086@cgmh.org.tw (K.-H.T.); glorydmoment@gmail.com (P.-Y.F.); dryctian@adm.cgmh.org.tw (Y.-C.T.); cwyang@cgmh.org.tw (C.-W.Y.); fangjits@cgmh.org.tw (J.-T.F.); 2 Transplantation Immunology Lab, Chang-Gung Memorial Hospital, Taoyuan 333, Taiwan; 3 Department of Pathology, Chang-Gung Memorial Hospital, Taoyuan 333, Taiwan; 8902028@cgmh.org.tw (T.-D.C.); s12126@cgmh.org.tw (W.-Y.C.); 4 College of Medicine, Chang-Gang University, Taoyuan 333, Taiwan; clku@cgu.edu.tw; 5 Division of Allergy, Asthma and Rheumatology, Department of Pediatrics, Chang-Gung Memorial Hospital, Taoyuan 333, Taiwan; joywucgu@hotmail.com; 6 Advanced Immunology Lab, Chang-Gung Memorial Hospital, Taoyuan 333, Taiwan

**Keywords:** TAFRO syndrome, idiopathic multicentric Castleman disease (MCD), IL-6, VEGF, renal thrombotic microangiopathy

## Abstract

TAFRO syndrome is an extremely rare form of idiopathic MCD, characterized by thrombocytopenia, anasarca, fever, reticulin fibrosis on bone marrow biopsy, and organomegaly. Like idiopathic MCD, renal involvement is also a common presentation in patients with TAFRO syndrome. Furthermore, membranoproliferative glomerulonephritis (MPGN)-like injury and thrombotic microangiopathy (TMA) are the most reported histopathologic findings of renal biopsy. Several molecular mechanisms have been previously postulated in order to explain the TAFRO syndrome symptoms, including abnormal production of interleukin-6 (IL-6), vascular endothelial growth factor (VEGF), etc. The role of these cytokines in renal injury, however, is not well understood. The aim of this review article is to summarize the latest knowledge of molecular mechanisms behind the TAFRO syndrome and their potential role in renal damage.

## 1. Introduction

Castleman disease is a rare lymphoproliferative disorder that encompasses specific histopathological features of lymphadenopathy. Depending on the area involved, Castleman disease can be categorized into two groups [1]. Unicentric Castleman disease (UCD) indicates the involvement of only one lymph node with typical pathological features and is less accompanied by constitutional symptoms. Multicentric Castleman disease (MCD), however, involves several lymph nodes. Multicentric Castleman disease, regardless of subtype, is frequently associated with systemic involvement. Elevated serum interleukin-6 (IL-6) and vascular endothelial growth factor (VEGF) are frequently reported to account for some of its symptoms. Renal involvement is also a common manifestation in patients with multicentric Castleman disease. Most cases receiving renal biopsy are presented with thrombotic microangiopathy (TMA) or membranoproliferative glomerulonephritis (MPGN)-like lesions [2]. Membranous nephropathy, however, is occasionally noted in some patients [3].

**Figure 1:**
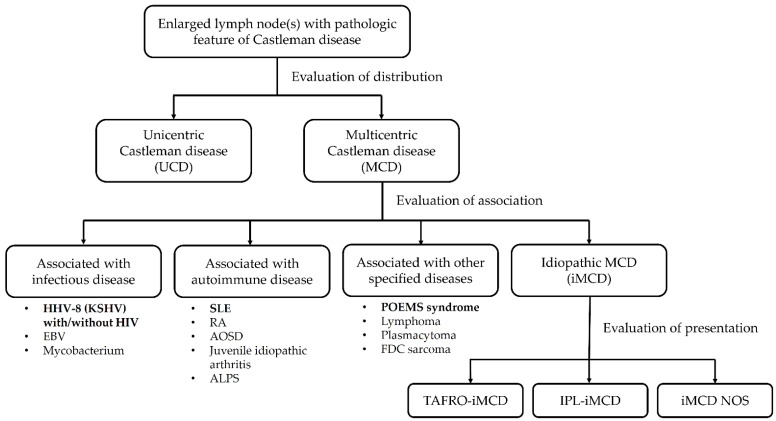
Diagnosis algorithm of Castleman disease. Abbreviation: ALPS, autoimmune lymphoproliferative syndrome; AOSD, adult-onset Still’s disease; EBV, Epstein-Barr virus; FDC sarcoma, follicular dendritic cell sarcoma; HHV-8, human herpesvirus 8; HIV, human immunodeficiency virus; IPL, idiopathic plasmacytic lymphadenopathy; KSHV, Kaposi’s sarcoma-associated herpesvirus; RA, rheumatoid arthritis; SLE, systemic lupus erythematosus.

Recently, one unique subgroup of MCD has been postulated. TAFRO syndrome was first described in 2010 [4]. A middle-aged Japanese male patient was diagnosed with MCD, which was accompanied by thrombocytopenia (T), anasarca (A), fever (F), reticulin fibrosis (R) on bone marrow biopsy, and organomegaly (O). After the first case was described, an increasing number of cases of TAFRO syndrome have been reported, mostly in Japan. According to associated diseases, MCD could be further categorized into idiopathic multicentric Castleman disease (iMCD), human herpes virus-8 (HHV-8)-associated, POEMS (polyneuropathy, organomegaly, endocrinopathy, M protein, and skin change) syndrome-associated, and some other associated diseases. iMCD are further classified into TAFRO-iMCD, IPL (idiopathic plasmacytic lymphadenopathy)-iMCD, and iMCD-NOS (not otherwise specified) IPL-iMCD, and KSVH-MCD according to presentations (Figure 1). Unlike traditional idiopathic MCDs presenting with thrombocytopenia or thrombocytosis and polyclonal hypergammaglobulinemia, TAFRO syndrome is exclusively associated with thrombocytopenia and normo-gammaglobulinemia [5]. Current diagnostic criteria for idiopathic Castleman disease and TAFRO syndrome are listed in Table 1. Based on the discrepancy in platelet count and total immunoglobulin G (IgG) levels, TAFRO syndrome is seen as a different disease from idiopathic MCD in some studies, although there are many shared clinical features between the TAFRO syndrome and idiopathic MCD [6]. Renal involvement is also frequently found in patients with TAFRO syndrome. Pathologic findings on renal biopsy, however, have not been examined in patients with TAFRO syndrome [7]. Resembling idiopathic MCD, thrombotic microangiopathy, and MPGN-like lesions are still the most common histological findings on renal biopsy in patients with TAFRO. The mechanism of renal involvement in those patients has not been explained to date.

## 2. Pathophysiologic Mechanisms of Castleman Disease and TAFRO Syndrome

Castleman disease is characterized by enlarged lymph nodes with specific histological features. It represents the histologic spectrum of two ends: hyaline-vascular type and plasma cell type [6]. The hyaline-vascular type is characterized by regressed geminal centers and prominence of follicular dendritic cells (FDCs), and the plasma cell type is characterized by hyperplastic germinal centers with profound plasma cell infiltration. Both histologic subtypes express varying degrees of vascularity, with vessels penetrating the geminal centers. Mixed pathology with combined hyaline vascular and plasmacytosis has also been reported. Regardless of the histologic subtypes, Castleman disease is exclusively associated with the dysregulated proliferation of specific lymphocytes. Plasma cells, by definition, are mature B cells able to produce antibodies and various cytokines involved in the inflammatory process. Because of malignant plasmacytoma, a cluster of plasma cells may exhibit dysregulated functionality, regardless of cytokines or antibodies. Follicular dendritic cells play a critical role in B cell activation and affinity maturation of antibodies [8]. In cases of follicular dendritic sarcoma, autoantibody production accompanied by paraneoplastic pemphigus is frequently reported [9]. Thus, the key manifestations of Castleman disease rely on the proliferation of specific lymphocytes, in addition to specific histological features and products of these lymphocytes, which account for clinical symptoms. The etiology of the lymphocyte proliferation in Castleman disease is not clear. Unlike in the POEMS syndrome, lymphoma, and myeloma, the absence of aberrant mitotic activity and clonal expansion mark these cells as relatively benign. The Castleman Disease Collaborative Network (CDCN) has proposed four candidate etiologic drivers, including self-reactive antibodies, germline mutations in genes regulating inflammation, acquired oncogenic mutations, and pathogen infections [10]. Further research is needed to shed light on the exact etiology of the disease.

Certain cytokines have been reported in cases of Castleman disease, including IL-6, VEGF, tumor necrosis factor-α(TNF-α), IL-1, and IL-8 [10,11]. Among these cytokines, IL-6 seems to play a major role in the pathophysiological mechanism behind the disease [12]. IL-6 is a well-known acute-phase protein of sepsis [13]. In the field of autoimmune diseases, such as systemic lupus erythematosus and rheumatoid arthritis, elevated serum IL-6 is frequently found, and it is possibly responsible for multiple systemic symptoms. When IL-6 is increasingly secreted in the circulation, it triggers many physical responses to several organs and systems. IL-6 acts on hepatocytes and induces increased production of several acute-phase proteins, including C-reactive protein (CRP), serum amyloid A (SAA), fibrinogen, and hepcidin, which account for amyloidosis, hypercoagulation, and anemia of inflammation. IL-6 could also drive the production of VEGF involved in angiogenesis in certain cancers [14,15,16]. IL-6 also influences the differentiation of primed CD4 naïve T cells. IL-6 promotes the differentiation of Th17 cells but inhibits the differentiation of Treg cells. Th17/Treg imbalance from the IL-6 effect results in autoimmunity and chronic inflammatory processes. Breakdown of immune tolerance may occur along with overproduction of autoantibodies [17].

Elevated serum IL-6 levels are largely found in patients with idiopathic multicentric Castleman disease [18]. Increased IL-6 expression is also found in lymph nodes involved in the disease [19]. Serum IL-6 and CRP concentrations drop rapidly after resection of lymphadenopathy in patients with unicentric Castleman disease, which brings relief of its clinical symptoms [20]. Considering the downstream effect of IL-6 on hepatocyte stimulation [13], serum CRP concentration is seen as a biomarker of disease activity in idiopathic multicentric Castleman disease. After initiation of treatment for idiopathic MCD, including glucocorticoid, cyclosporine, anti-CD20 antibody, and anti-IL6 receptor antibody, clinical symptoms tend to improve, which is accompanied by a decline in the serum CRP concentration [21,22,23]. Thus, IL-6 is a possible therapeutic target in cases of idiopathic MCD.

TAFRO syndrome is a unique disease characterized by systemic inflammation. The Castleman disease-like feature of histologic findings of lymph node biopsy is the cardinal manifestation of TAFRO syndrome [24]. This explains why TAFRO syndrome is seen as a special subtype of multicentric Castleman disease, although some studies claim that lymphadenopathy with Castleman disease-like features is not necessary for the diagnosis of TAFRO syndrome [25]. Clinical presentations of multicentric Castleman disease vary widely. Altered platelet count, including thrombocytopenia and thrombocytosis, can be found in patients with idiopathic MCDs [5]. Compared to the non-TAFRO idiopathic MCDs, TAFRO syndrome is associated exclusively with thrombocytopenia. In addition, hypergammaglobulinemia excludes the diagnosis of the TAFRO syndrome (normo-gammaglobulinemia). Based on certain shared features and some clinical discrepancies, it is believed that non-TAFRO idiopathic MCD and TAFRO syndrome have similar pathophysiological mechanisms. The role of IL-6 in patients with TAFRO syndrome is not well known. Elevated serum IL-6 levels, however, are still frequently found in patients with TAFRO syndrome [26]. The clinical response of targeted therapy for TAFRO syndrome is also closely related to serum IL-6 and CRP concentrations [27]. Elevated serum IL-6, however, is associated with thrombocytosis rather than thrombocytopenia [28]. In addition, there is no direct evidence that IL-6 promotes reticulin fibrosis and organomegaly. There are still unknown factors, on top of IL-6, that determine the presentation of non-TAFRO idiopathic MCD or TAFRO syndrome.

The autoimmune features of Castleman disease have been frequently mentioned in previous studies. Castleman disease frequently mimics systemic lupus erythematosus (SLE) in terms of the production of certain autoantibodies [29]. Pemphigus, with the production of anti-BP180 autoantibody, was reported in a patient with Castleman disease [30]. In cases of HHV-8 associated with Castleman disease, acquired hemophilia secondary to anti-factor VIII autoantibodies was also reported [31]. TAFRO syndrome is characterized by many clinical symptoms with a key manifestation of thrombocytopenia. Antiplatelet autoantibodies are frequently found, which account for remarkable thrombocytopenia [32,33]. Considering the histologic features of lymphadenopathy of Castleman disease, the prominence of plasma cells or follicular dendritic cells may lead to the overproduction of various autoantibodies. Some of these studies claimed that clinical features of Castleman disease and TAFRO syndrome are mostly determined by the autoantibodies and cytokines produced by specific clones of B lymphocytes and that TAFRO syndrome represents a unique group of idiopathic MCD with shared autoimmune features.

## 3. Renal Thrombotic Microangiopathy and Its Proposed Mechanism

“Thrombotic microangiopathy (TMA)” is a confusing term due to its historical evolution [34]. Originally, thrombotic microangiopathy was a pathological description characterized by endothelial injury and thrombi formation of the microvasculature, especially in the kidney [35]. The term simply depicts the morphologic change under a light microscope, regardless of the pathophysiological mechanism. Nowadays, thrombotic microangiopathy is sometimes used to describe a syndrome characterized by clinical features of thrombocytopenia, microangiopathic hemolytic anemia (MAHA), and organ injury [36]. The confusion stems from the similarity between thrombotic thrombocytopenic purpura (TTP) and hemolytic uremic syndrome (HUS).

Thrombotic thrombocytopenic purpura, hereditary or acquired, is secondary to deficiency of ADAMTS13 (a disintegrin and metalloproteinase with a thrombospondin type 1 motif, member 13), which causes the formation of large von Willebrand factor (vWF) multimers and platelet aggregation. Platelet-rich thrombi result in thrombocytopenia, microangiopathic hemolytic anemia (MAHA), and secondary endothelial injury [37]. Renal involvement is sometimes seen in patients with TTP, but its degree is often mild [38]. Under light microscopy, platelet-rich thrombi of the hilum or capillary loop are more frequent than endothelial injury. On the other hand, HUS has similar clinical and laboratory findings, but the pathologic features of HUS differ from those of TTP. HUS, typical or atypical, is caused by endothelial damage caused by various mechanisms, including direct injury from toxins, drugs, or antibodies and indirect injury from uncontrolled activation of the complement system. The leading feature of HUS is endothelial swelling of the involved organ, especially the kidney. The prothrombotic state is secondary to endothelial injury of the involved vasculature [39].

Although attributed to different molecular mechanisms, TTP and HUS still overlap with each other in terms of clinical presentations. Nowadays, many diseases resemble TTP or HUS, with presentations of MAHA and/or pathological features of endothelial injury. All these diseases are collectively referred to as “TMA syndromes” [40]. Some of these diseases have little pathological evidence of endothelial injury (e.g., TTP), and others have no clinical or laboratory features of MAHA (e.g., anti-VEGF-related renal TMA [41]).

The kidney is the major organ involved in patients with TMA syndromes. It is not yet clear why renal vasculature is so vulnerable to endothelial injury. The unique architecture of glomerular capillaries may account for this susceptibility. Glomerular capillaries are composed of thin and loose fenestrated vascular endothelium. Glycocalyx synthesized by the vascular endothelium forms a charge-selective filtration barrier of the glomerular basement membrane. VEGF is the major cytokine that maintains the integrity and functionality of the vascular endothelium [42]. In vitro evidence suggests that in glomerular capillaries, VEGF induces the fenestrate formation of the vascular endothelium. The glomerular endothelium has surface receptors for the vascular endothelial growth factor (VEGF-R). The primary source of VEGF nursing the glomerular endothelial cell are visceral epithelial cells, podocytes, rather than the circulating VEGF [43]. Breakdown of VEGF backflow from podocytes to endothelial cells could result in endothelial damage of glomerular capillary [44]. This unique feature is only present in the renal vasculature. Although the mechanism has not been clarified yet, this may provide a possible explanation of renal susceptibility to endothelial injury.

We summarized the diseases known as TMA syndrome and the proposed mechanism of renal thrombotic microangiopathy (Table 2). As mentioned above, TMA syndrome is composed of various disease entities. According to the mechanism, the TMA syndrome has been divided into the following categories: activation of the coagulation system, direct endothelial injury, indirect endothelial injury, and miscellaneous. The first category comprises diseases involving activation of the coagulation system along with secondary endothelial injury. A suitable example is thrombotic thrombocytopenia (TTP), in its genetic or acquired form, which causes large vWF multimer formation and platelet-rich thrombosis. Microthrombi formation and activation of the coagulation cascade resulting in secondary endothelial injury. Hemolytic uremic syndrome (HUS) with genetic mutation of *MMACHC* [45], THBD [46], and DKGE [47] is characterized by similar activation of the coagulation system.

The second category is composed of diseases involving direct endothelial injury and prothrombotic state. Shiga toxin-producing Escherichia coli (STEC)-related hemolytic uremic syndrome, or “typical” HUS, demonstrates toxin-induced direct endothelial damage [48], followed by the systemic manifestation. Autoimmune diseases, such as systemic lupus erythematosus (SLE) [49], anti-phospholipid syndrome (APS) [50], and systemic sclerosis (SSc) [51], can produce autoantibodies that have binding affinity to endothelial cells and may cause tremendous damage. Calcineurin inhibitors (CNIs) have numerous effects on the vascular endothelium [52]. Inhibition of prostacyclin synthesis and formation of activated protein C are believed to be major mechanisms of CNI-related endothelial injury [53]. Radiation therapy used in cancer treatment is also associated with thrombotic microangiopathy. Direct endothelial injury from radiation is frequently reported [54].

The third category covers the hemolytic uremic syndrome with defects in complement regulation and several diseases associated with the blockade of VEGF. Complement-mediated hemolytic uremic syndrome is an emerging disease that involves aberrant activation of alternative pathways of the complement system [39]. Currently, mutations in several genes encoding complement factors, including CFH, CFI, MCP, and CFB, are postulated in the pathogenesis of complement-mediated HUS. Autoantibodies against these complement factors also have similar effects. Both genetic mutations and autoantibodies result in uncontrolled activation of the complement system, followed by endothelial injury and prothrombotic status. VEGF blockade, including the presence of VEGF antagonist or inhibition of the VEGF signaling pathway, often causes a similar endothelial injury, especially in the kidney. As mentioned above, VEGF nurses the vascular endothelial cells of the capillary loop. Deprivation of local VEGF expression causes subsequent endothelial swelling, followed by the histological feature of thrombotic microangiopathy [44].

There are still several diseases classified as TMA syndromes without association to the abovementioned mechanisms or related to more complex mechanisms explainable by multi-hit theories.

Sepsis [55] or cancer-related [56] disseminated intravascular coagulation (DIC), HELLP syndrome (composed of hemolytic anemia, elevated liver enzymes, and low platelet count) [57], malignant hypertension [58], are mentioned as TMA syndromes in the literature. Idiopathic MCD and TAFRO syndrome may have some similar or shared features with these diseases. The mechanism of idiopathic MCD or TAFRO syndrome causing these clinical presentations, however, has not been explained yet.

## 4. From TAFRO Syndrome to Renal Thrombotic Microangiopathy

Thrombocytopenia is a typical presentation of the TAFRO syndrome. According to the definition by Iwaki [26] and Masaki [24], the presence of thrombocytopenia fulfills one of the major diagnostic criteria. Microangiopathic hemolytic anemia or schistocytosis, however, have never been mentioned in previous studies. Renal involvement is frequent in these patients, but it cannot guarantee the diagnosis. In addition, kidney injury in patients with TAFRO syndrome varies from thrombotic microangiopathy to membranoproliferative glomerulonephritis (MPGN). Therefore, it is suggested that there are factors other than IL-6 and VEGF, which dominate the glomerular injury.

Elevated serum VEGF has frequently been reported in cases of idiopathic MCD and TAFRO syndrome. Considering the current knowledge on the role of VEGF in thrombotic microangiopathy, elevated serum VEGF hardly explains endothelial injury because of the nursing effect of VEGF on vascular endothelial cells. Although elevated serum VEGF has been reported in some cancer studies [59], no clear evidence supports the relationship between serum VEGF and cancer-associated renal thrombotic microangiopathy. In other aspects, podocytes are believed to be the origin of VEGF in the glomerular endothelial cells. Podocytes produce VEGF and create a concentration gradient of VEGF across the glomerular basement membrane, causing VEGF diffusion backflow opposite of glomerular filtration [41]. It is questionable whether the polarity of VEGF-R expression in vascular endothelial cells exists. The effect of elevated serum VEGF levels on vascular endothelial cells is also unclear.

**Figure 2:**
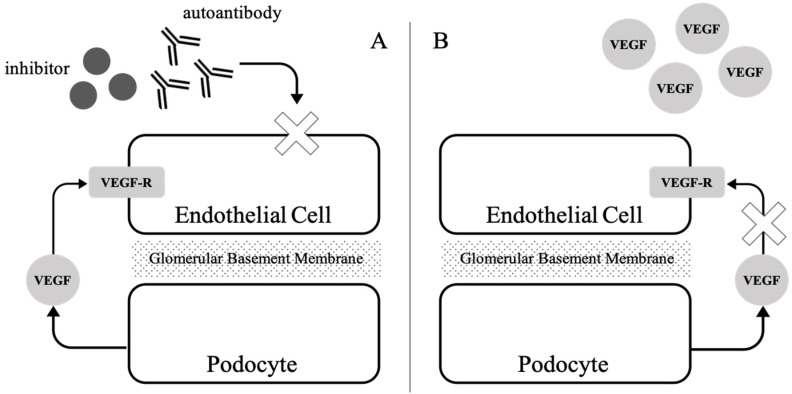
Proposed mechanism of renal thrombotic microangiopathy in a patient with TAFRO syndrome. (**A**). Circulating autoantibodies or cytokines produced by specific (**B**) lymphocyte clones within lymphadenopathy interfere with VEGF diffusion flux or directly act on vascular endothelial cells causing damage, (**B**). Elevated serum VEGF breaks the concentration gradient of VEGF across the glomerular basement membrane, impeding the nursing effect of VEGF produced by podocytes.

According to the current knowledge of idiopathic MCD and TAFRO syndrome, there is no evidence of activation of the coagulation system and complement system. No studies have provided clear evidence of the association between elevated serum IL-6 levels and thrombotic microangiopathy. Considering the autoimmune features of Castleman disease and the unique nursing role of VEGF in vascular endothelial cells, we proposed two hypotheses explaining the mechanism of renal thrombotic microangiopathy in the Castleman disease, especially TAFRO syndrome (Figure 2). First, specific B lymphocytes show clonal expansion and produce autoantibodies or inhibitors, which interfere with VEGF diffusion flux or directly act on vascular endothelial cells, followed by endothelial injury. Second, increased serum VEGF diminishes the concentration gradient of VEGF produced by the podocytes, which impedes the nursing effect and causes endothelial injury. Both hypotheses need to be confirmed by further studies. Considering the hypothesis of autoantibodies, high-throughput assays involving autoantibody screening from a patient’s serum may prove it. Considering the hypothesis of the VEGF concentration gradient breakdown, comprehensive in vitro cell line-based studies and animal models are warranted to explore it.

## 5. Conclusions and Future Directions

Idiopathic MCD is an uncommon disorder with unique clinical features. TAFRO syndrome is an extremely rare subtype of idiopathic MCD with a specific clinical presentation. The diagnosis and management of these disorders are challenging. The features of these disorders are quite complicated, from lymphadenopathies, cytokines, and autoantibodies from specific B cell clones, constitutional symptoms, and end-organ damage. Comprehensive and collaborative research work could provide an opportunity to clarify the pathophysiologic mechanism of these unique diseases. Furthermore, knowledge of these disorders may help clinicians to elucidate the pathogenesis of other lymphoproliferative disorders and the mechanism of breakdown of B cell tolerance. Further knowledge about these diseases will provide more precise treatment for these patients (Table 3)

## Figures and Tables

**Table 1:** Comparison of diagnostic criteria of the idiopathic multicentric Castleman disease and TAFRO syndrome.

2015 Iwaki Criteria for TAFRO-iMCD	2015 Masaki Criteria for TAFRO Syndrome	2017 International Consensus Criteria for iMCD
**Histopathologic criteria** (prerequisite) • Compatible with pathological findings of lymph nodes as TAFRO-iMCD • Negative LANA-1 for HHV-8 **Major criteria** (all required) • Presents 3 of 5 TAFRO symptoms • - Thrombocytopenia - Anasarca - Fever - Reticulin fibrosis - Organomegaly • Absence of hypergammaglobulinemia • Small volume lymphadenopathy **Minor criteria** (1 or more required) • Hyper/normoplasia of megakaryocytes in the bone marrow • High levels of serum ALP without markedly elevated serum transaminase	**Major categories** (all required) • Anasarca, including pleural effusion, ascites, and general edema • Thrombocytopenia defined as a pre-treatment platelet count ≤ 100,000/μL • Systemic inflammation defined as fever of unknown etiology above 37.5 °C and/or serum CRP ≥ 2 mg/dL **Minor categories** (need 2 or more) • Castleman’s disease-like features on lymph node biopsy • Reticulin myelofibrosis and/or increased number of megakaryocytes in the bone marrow • Mild organomegaly, including hepatomegaly, splenomegaly, and lymphadenopathy • Progressive renal insufficiency **Diseases to be excluded**: • Malignancy, autoimmune disorders, infectious disorders, POEMS syndrome, IgG4-related disease, cirrhosis, and TTP/HUS	**Major criteria** (both required) • Histopathologic lymph node features consistent with the iMCD spectrum • Enlarged lymph nodes (≥1 cm in short-axis diameter) in ≥2 lymph node stations **Minor criteria** (2 or more required with at least 1 laboratory criterion) *Laboratory criteria* • Elevated CRP (>10 mg/L) or ESR (>15 mm/h) • Anemia (hemoglobin < 12.5 g/dL for males, hemoglobin <11.5 g/dL for females) • Thrombocytopenia (platelet count <150 k/mL) or thrombocytosis (platelet count >400 k/mL) • Hypoalbuminemia (albumin <3.5 g/dL) • Renal dysfunction (eGFR <60 mL/min/1.73 m^2^) or proteinuria (total protein 150 mg/24 h or 10 mg/100 mL) • Polyclonal hypergammaglobulinemia (total γ-globulin or IgG > 1700 mg/dL) *Clinical criteria* • Constitutional symptoms: night sweats, fever (>38 °C), weight loss, or fatigue (≥2 CTCAE lymphoma score for B-symptoms) • Large spleen and/or liver • Fluid accumulation: edema, anasarca, ascites, or pleural effusion • Eruptive cherry hemangiomatosis or violaceous papules • Lymphocytic interstitial pneumonitis **Exclusion criteria** • Infection-related disorder, autoimmune/autoinflammatory disease, malignant/lymphoproliferative disorders

Abbreviations: ALP, alkaline phosphatase; CRP, C-reactive protein; CTCAE, Common Terminology Criteria for Adverse Events; eGFR, estimated glomerular filtration rate; ESR, erythrocyte sedimentation rate; HHV, human herpesvirus; HUS, hemolytic uremic syndrome; iMCD, idiopathic multicentric Castleman disease; IgG, immunoglobulin G; LANA, latency-associated nuclear antigen; TTP, thrombotic thrombocytopenic purpura.2. Pathophysiologic mechanisms of Castleman disease and TAFRO syndrome.

**Table 2:** Categories of renal thrombotic microangiopathy based on the mechanism.

Disease Category	Major Mechanism of Renal Injury
**Activation of coagulation system with secondary endothelial injury**	
∙ Genetic or acquired TTP	ADAMTS13 deficiency from gene mutation or autoantibody causes large vWF multimers formation and platelet-rich thrombosis
∙ HUS with a defect in the cobalamin and coagulation pathway	Genetic mutation of *MMACHC*, *THBD*, *DKGE* affect the regulation of the coagulation system
∙ Drugs: clopidogrel, ticlopidine	Associated with the presence of anti-ADAMTS13 autoantibodies or inhibitors
**Direct endothelial injury with prothrombotic state**	
∙ Toxin-related HUS: Shiga-like toxin, neuraminidases	Shiga-like toxins bind to Gb3 receptors of endothelial cells and directly cause damage; neuraminidases expose cryptic antigens of endothelial cells and elicit immunologic damage
∙ Autoantibody: SLE, APS, systemic sclerosis	Antiphospholipid antibody or anti-endothelial antibody directly cause endothelial injury
∙ Drug: calcineurin inhibitor (CNI)	CNI reduce the prostacyclin synthesis and formation of activated protein C, which causes direct endothelial damage
Radiation	Direct endothelial injury from radiation effect
**Indirect endothelial injury with prothrombotic state**	
∙ HUS with a defect in complement regulation	Gene mutation or autoantibody-inhibition of complement regulatory factors, e.g., *CFH*, *CFI*, *MCP*, *CFB*, result in uncontrolled activation of the alternative complement pathway, followed by endothelial injury and prothrombotic state
∙ VEGF blockade: preeclampsia and eclampsia	Overexpression of sFlt1 from the placenta acts as an antagonist of VEGF, causing endothelial injury
∙ VEGF blockade: anti-VEGF, tyrosine kinase inhibitor	Inhibition of the VEGF pathway directly causes endothelial swelling and disruption of cell integrity
∙ VEGF blockade: mTOR inhibitor	mTOR regulates the intracellular pathway of VEGF production. mTOR inhibitor results in a decrease in VEGF production, followed by endothelial injury
**Miscellaneous**: DIC, neoplasm, HELLP syndrome, malignant hypertension, idiopathic multicentric Castleman disease, TAFRO syndrome.	

Abbreviations: ADAMTS13, a disintegrin and metalloproteinase with a thrombospondin type 1 motif, member 13; APS, antiphospholipid syndrome; CFB, complement factor B; CFH, complement factor H; CFH, complement factor I; DGKE, diacylglycerol kinase ε; Gb3, globotriaosylceramide; HUS, hemolytic uremic syndrome; MCP, membrane cofactor protein; MMACHC, methylmalonic aciduria, and homocystinuria type C protein; mTOR, mammalian target of rapamycin; sFlt1, soluble fms-like tyrosine kinase-1; SLE, systemic lupus erythematosus; THBD, thrombomodulin; TTP, thrombotic thrombocytopenic purpura; VEGF, vascular endothelial growth factor; vWF, von Willebrand factor.

**Table 3:** Current treatment options of Castleman disease and associated disorders.

Disease Category	Possible Treatment
iMCD or TAFRO-iMCD	Anti-IL6 (siltuximab, tocilizumab) Corticosteroid Anti-CD20 (rituximab) Proteasome inhibitors (Bortezomib) IVIG Calcineurin inhibitors (cyclosporin) mTOR inhibitors (sirolimus) Immunomodulatory drugs (Thalidomide, Lenalidomide) Chemotherapy (R-CHOP, R-CVP) Autologous stem cell transplantation
HHV-8 associated MCD	If HIV exists, provide antiviral agents. Anti-CD20 (rituximab) Interferon Chemotherapy: Etoposide, Doxorubicin.
POEMS associated MCD	If no bone lesion, treated as iMCD. If bone lesion exists, treated as plasmacytoma or multiple myeloma.
Other association	Treat as associated disease

Modified from Dispenzieri et al. [60]. Abbreviation: HHV-8, human herpesvirus 8; HIV, human immunodeficiency virus; IL-6, interleukin-6; iMCD, idiopathic Castleman disease; IVIG, intravenous immunoglobulin; mTOR, mammalian target of rapamycin; R-CHOP, rituximab, cyclophosphamide, doxorubicin, vincristine, prednisone; R-CVP, rituximab, cyclophosphamide, vincristine, prednisone.
